# IsoPlotter^+^: A Tool for Studying the Compositional Architecture of Genomes

**DOI:** 10.1155/2013/725434

**Published:** 2013-04-18

**Authors:** Eran Elhaik, Dan Graur

**Affiliations:** ^1^Department of Mental Health, Johns Hopkins University Bloomberg School of Public Health, Baltimore, MD 21205, USA; ^2^Department of Biology and Biochemistry, University of Houston, Houston, TX 77204-5001, USA

## Abstract

Eukaryotic genomes, particularly animal genomes, have a complex, nonuniform, and nonrandom internal compositional organization. The compositional organization of animal genomes can be described as a mosaic of discrete genomic regions, called “compositional domains,” each with a distinct GC content that significantly differs from those of its upstream and downstream neighboring domains. A typical animal genome consists of a mixture of compositionally homogeneous and nonhomogeneous domains of varying lengths and nucleotide compositions that are interspersed with one another. We have devised IsoPlotter, an unbiased segmentation algorithm for inferring the compositional organization of genomes. IsoPlotter has become an indispensable tool for describing genomic composition and has been used in the analysis of more than a dozen genomes. Applications include describing new genomes, correlating domain composition with gene composition and their density, studying the evolution of genomes, testing phylogenomic hypotheses, and detect regions of potential interbreeding between human and extinct hominines. To extend the use of IsoPlotter, we designed a completely automated pipeline, called IsoPlotter^+^ to carry out all segmentation analyses, including graphical display, and built a repository for compositional domain maps of all fully sequenced vertebrate and invertebrate genomes. The IsoPlotter^+^ pipeline and repository offer a comprehensive solution to the study of genome compositional architecture. Here, we demonstrate IsoPlotter^+^ by applying it to human and insect genomes. The computational tools and data repository are available online.

## 1. Introduction 

While the genome sizes of multicellular eukaryotes are generally larger and more variable in length than those of prokaryotes, guanine and cytosine (GC) content exhibits a much smaller variation in eukaryotes than in prokaryotes. In particular, vertebrate genomes show quite a uniform GC content, distributing over a very narrow range from about 40% to 45% [1]. Despite the uniformity of their genomic GC content, vertebrate genomes have a much more complex compositional organization than prokaryotic genomes. Recent studies have shown that this narrow distribution cloaks a complex mosaic of homogeneous and nonhomogeneous compositional domains whose sizes range from 3 kilobases (kb) to more than 10 Mega bases (Mb) and whose GC contents range from ~7% to ~72% (e.g., [2, 3]). Molecular evolutionists have had a long-standing interest in deciphering the internal compositional organization of genomes, describing their structure, and identifying the nature of the forces driving base compositional variation (e.g., [4–9]). Thus far, the “compositional domain model” [3, 10] was validated in both vertebrate and invertebrate genomes [2, 11–19], opening up new venues of research in a field of study that was stuck in a rut of semantic and methodological tediousness [4, 10, 20] for over four decades.

Before the advent of complete genomes that can be analyzed with genomic segmentation methods, compositional domains were inferred by using indirect proxies such as the GC content of third codon positions (GC3). The GC3 approach was shown to be a poor stand-in for the composition of long genomic regions [20–22]. Unfortunately, sequence segmentation approaches differed greatly in their performances and often performed poorly because of the arbitrary choice of input variables [3, 10]. Through comparison of performances against benchmark simulations, Elhaik et al. [10] identified a class of recursive segmentation algorithms based on the Jensen-Shannon divergence (*D*_JS_) that outperformed all other methods. However, one main difficulty with these algorithms concerned the criteria for halting the segmentation. To address these issues, we developed IsoPlotter, a parameter-free segmentation algorithm that overcomes such biases by using a simple dynamic halting criterion and tests the homogeneity of the inferred domains against the compositional homogeneity of the chromosome in which they are found [3]. 

**Figure 1:**
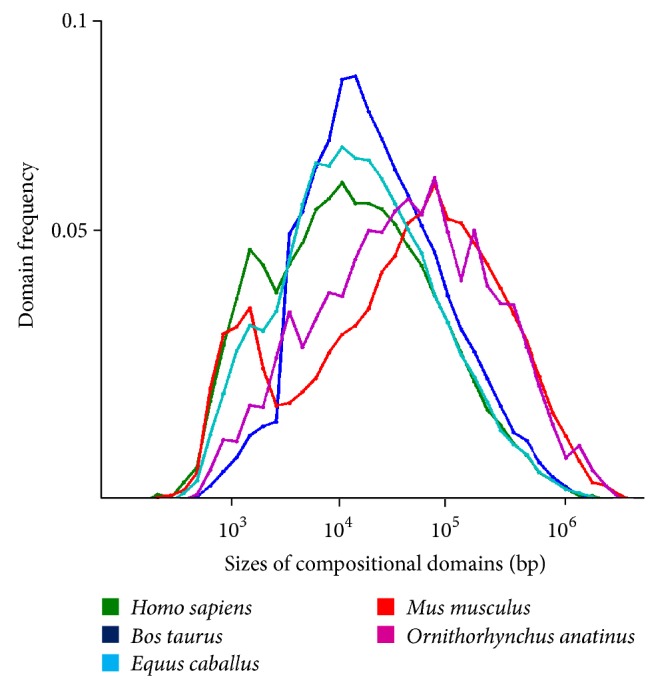
The frequency of compositional domains for five taxa.

**Figure 2:**
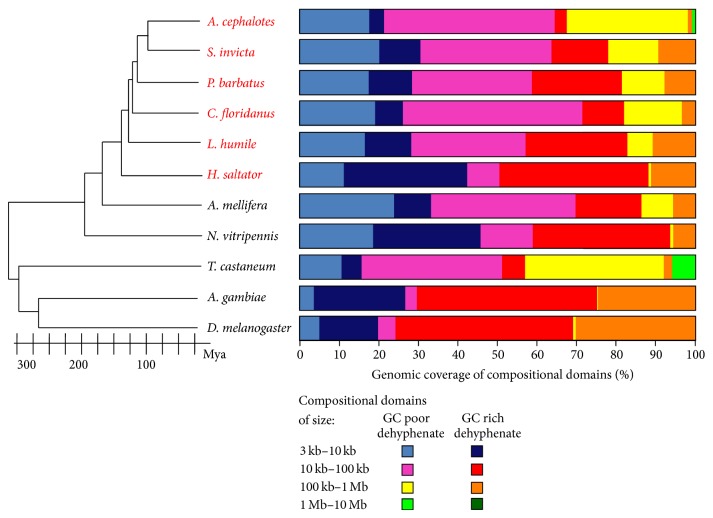
Compositional organization of eleven insect genomes including six newly sequenced ants (red). Compositional domains were classified as GC poor or GC rich based on the mean GC content of all insect genomes (37.62%) and further divided by size. The similarity in genome compositional organization can be compared to the estimated phylogenetic tree constructed based on recent publications [11–13].

Our *D*_JS_-based algorithms and IsoPlotter have been applied to numerous genomic analyses including honeybee, sea urchin, red-flour beetle, cow, *Nasonia*, body louse, and several ant genomes [2, 11–19]. These studies have demonstrated the applicability of our approach and the robustness of the compositional domain model. Different applications of IsoPlotter include describing new genomes (e.g., [17]), testing the quality of new genome builds by comparing their domain size distributions (Figure 1) (e.g., [2]), studying the distribution of genes with respect to GC content (e.g., [16]), studying methylation patterns along domains (e.g., [11]), carrying comparative analyses at the fine-genomic level (Figure 2) (e.g., [13]), and testing phylogenomic analyses that assess relatedness between species based on the similarity of their genomic landscapes, such as shown in Figure 2. Some of these applications utilized IsoPlotter's ability to identify cluster of homogeneous regions to detect regions of potential interbreeding between human and extinct hominines, such as Neanderthal and Denisovan [23]. Further evolutionary applications include identifying the evolutionary mechanisms that shaped the compositional architecture and the mechanisms that drive the evolution of genomes.

Despite its premise, few limitations hindered broader usability of the original IsoPlotter tool. IsoPlotter neither supports multiple species analyses nor handles *N*s regions. Due to the additional steps of preparing the input and interpreting the output files, IsoPlotter's usability was limited. Moreover, IsoPlotter's scripts could be executed only by MATLAB users.

The goal of this note is to address all these issues and announce a new IsoPlotter pipeline (IsoPlotter^+^) that carries all segmentation analyses in chromosomal and scaffold sequences and includes a graphical display for carrying out further analyses. The pipeline includes tools to correct for gaps in the genomic sequence and tests the homogeneity of compositional domains compared to the genomic region on which they reside, as described in Elhaik et al. [3]. We also built a repository for the compositional domain maps of all fully sequenced vertebrate and invertebrate genomes. The pipeline was implemented in MATLAB 7.5. The pipeline and repository are available at http://code.google.com/p/isoplotter/. In addition to MATLAB scripts, we provide executable files that can run with the freely available MATLAB MCRInstaller tool (see our user guide). We demonstrate the pipeline on the human and insect genomes.

## 2. Results and Discussion

### 2.1. A Repository for Compositional Domain Maps

We applied the IsoPlotter^+^ pipeline to all fully sequenced vertebrate and invertebrate genomes and built the most comprehensive online repository for compositional domain maps. The only other repository for compositional domains is available at http://bioinfo2.ugr.es/isochores/. This repository includes less than a dozen genomes and was last updated in 2007. Unfortunately, its results cannot be reproduced as the tool used to obtain these results no longer exists (see [10]). Domain compositional maps will be regularly updated as new genomes are sequenced or new builds are published.

### 2.2. The Human Genome

Previous estimations for the number of compositional domains in the human genome varied from ~3,200 long homogeneous domains (“isochoric”) [24] to ~32,000 [25] and ~47,000 [4] short and long domains. To demonstrate the use of the IsoPlotter^+^ pipeline we segmented the human genome (available in 24 FASTA files, one for each chromosome under the *Human* folder), using the MATLAB command

 RunIsoPlotterPipeline(‘C:*∖*Input*∖*Human*∖*', ‘C:*∖*Output*∖*Human*∖*')

**Figure 3:**
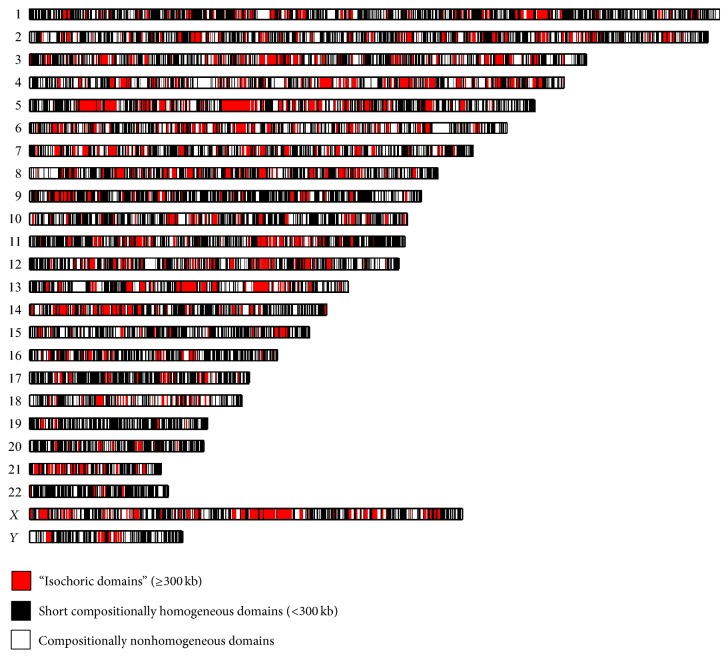
Ideogram of compositional domains in humans as inferred by IsoPlotter and mapped to chromosomes created using the PlotGenome.m script. The ideogram uncovers the compositional patterns of long compositionally homogeneous domains (“isochoric”), short compositionally homogeneous domains (<300 kb), and compositionally nonhomogeneous domains.

 The segmentation of the human genome was completed in less than an hour on a PC with dual-core and 3.5 GB of RAM creating a “seg_no_ns_H.txt” file, which contains the segmentation results. The results revealed a compositional substructure finer than has ever been described before. The human genome consists of approximately 120,000 domains, most of which are short, with only 7% of the compositional domains being longer than 300 kb ones. Nearly 70% of the domains were considered homogeneous with both homogeneous and nonhomogeneous domains exhibiting similar lengths and GC-content distributions. In terms of coverage, long homogeneous domains (>300 kb), also referred to as “isochoric domains,” cover less than 20% of the genome (Figure 3). By contrast, short compositionally homogeneous domains cover between 38% (chromosome 4) and 72% (chromosome 22) of the chromosomes. Similar results were obtained for the cow genome [2]. 

The IsoPlotter^+^ pipeline includes three ideograms that allow us to compare compositional patterns among chromosomes and describe different layers of the genome. These ideograms can be generated using the command

 PlotGenome(‘C:*∖*Output*∖*seg_no_ns_H.txt', ‘C:*∖*Output*∖*PlotGenome.tif')

 The first ideogram compares the genome distribution of homogeneous and nonhomogeneous domains. It shows that compositionally homogeneous domains cover between 62% (chromosome 4) and 78% (chromosome 11) of the chromosomes [3]. Dividing compositionally homogeneous domains into long (>300 kb) and short domains reveals that “isochoric domains” are heterogeneously distributed along chromosomes covering between 5% (chromosome 19) and 29% (chromosome 5) of the chromosomes (Figure 3). The third ideogram further classifies “isochoric domains” into low GC domains ranging from 20% to 40% (574 domains) and high GC domains ranging from 40% to 60% (245 domains) with a single rich GC domain (61%) in chromosome 16. These results indicate that 70% of all “isochoric domains” are AT rich, in contrast to the literature claims spanning a period of about 40 years (e.g., [24]).

### 2.3. Insect Genomes

We next demonstrate the use of the IsoPlotter^+^ pipeline by segmenting all ant scaffolds, using the MATLAB command

 RunIsoPlotterPipeline(‘C:*∖*Input*∖*Ant_species*∖*', ‘C:*∖*Output*∖*Ant_species*∖*')

 The composition and organization of the compositional domains were shaped by different evolutionary processes that either fused or broke down the domains. Identifying these forces may be possible using a comparative analysis of multiple related species. For example, we have recently shown that that unlike other hymenopterans, long domains have rapidly accumulated along the ant linage with the leaf-cutters Atta cephalotes and Acromyrmex echinatior having the largest domains among all fully sequenced insect genomes [19]. In Figure 2, we show the genomic coverage of compositional domains as a function of domain composition and length. Because the results for the ant genomes were obtained from scaffolds, caution is warranted in interpreting results regarding short domains (<10 kb) that skew the results. We thus focus our analysis on long domains (>100 kb) that are more robust to error [2]. Interestingly, long domains appear to accumulate rapidly over time in the ant genome with *H. saltator* having only a dozen long domains and *A. cephalotes* having almost three dozens. We also observed large shifts in genome composition. In dipterans, the vast majority of domains (92%) are GC rich, whereas in the red flour beetle (Tribolium castaneum) only 13% of the domains are GC rich. In ants, the proportion of GC-rich domains ranges from 8% (*A. cephalotes*) to 80% (*H. saltator*) with a mean of 41.62 ± 23.74. Such changes in the compositional organization of genomes over time hint at evolutionary processes that shape the compositional architecture (e.g., [26]). Studying the preference of genes to GC-poor or GC-rich compositional domains may help elucidate the mechanisms causing gene-containing regions to simultaneously alter their composition and to break or fuse domains. For example, we showed that in Hymenoptera, genes occur in more GC-poor than GC-rich regions (e.g., [11–13]). 

One way to study how such structures emerged during evolution would be to identify syntenic regions across species and compare their compositional domains. Related phylogeny-based analyses can be further carried with dedicated MATLAB packages, such as described by McLysaght et al. [27]. 

In summary, we have presented a fast new pipeline, tools, and repository suitable for investigating the evolution of genomes and comparing their compositional organizations to better understand the forces that shaped them. Because segmentation parameters are fixed, users can compare their results with those obtained by other users and use the built-in functions to visualize the genomic sequences. Our tools and repository are freely available at http://code.google.com/p/isoplotter/.

## 3. Materials and Methods

### 3.1. The IsoPlotter Segmentation Algorithms

IsoPlotter is a binary, recursive segmentation algorithm that splits a DNA sequence by finding a point that maximizes the difference in GC content between adjacent subsequences. The resulting subsequences are recursively segmented until the halting condition is satisfied [3]. 

### 3.2. The Homogeneity Test

When the segmentation is completed, a homogeneity test determines whether the inferred domains are compositionally homogeneous or nonhomogeneous based on their homogeneity relative to the chromosome or genomic region on which they reside [3]. 

### 3.3. Handling N Islands

Genomic sequences often include nucleotides marked as “*N*,” representing either gaps in the sequence or polymorphic nucleotides. The IsoPlotter^+^ pipeline maps the positions of all the *N* nucleotides and then performs the segmentation on sequence files without *N*s. After the segmentation is complete, the domain borders are adjusted based on the *N* mapping. *N*s regions that are sufficiently small (<50 kb) (set by default, but can be adjusted) are masked, whether larger *N*s regions split the domains. For example, a compositional domain of 200 kb and GC content of 35% harboring a sequence of 50 kb *N*s in its middle will be split into three sequences: 1–75 kb (GC = 35%), 75–125 (GC = 0), and 125–200 kb (GC = 35%). 

### 3.4. Plotting Tools

We designed tools to visualize the spatial distribution of GC content and compositional domains at the chromosomal and genomic levels and view different layers of the compositional organization of the genome. Tools and usages are outlined in the user manual.

### 3.5. The IsoPlotter^+^ Repository

We ran the IsoPlotter^+^ pipeline for all fully sequenced vertebrate and invertebrate genomes for which chromosomal data was available. The compositional domain coordinate, GC content, GC content standard deviation, and indication of homogeneity are available at our repository accessible at http://code.google.com/p/isoplotter/.
